# Effects of macroconsumers on benthic communities: Rapid increases in dry-season accrual of calcium in a tropical karst stream

**DOI:** 10.1371/journal.pone.0209102

**Published:** 2018-12-21

**Authors:** Elaine Cristina Corrêa, Fabio de Oliveira Roque, Ryan Michael Utz, Jonas de Sousa Correa, Franco Leandro de Souza, Alan Paul Covich

**Affiliations:** 1 Instituto de Biociências, Universidade Federal de Mato Grosso do Sul, Campo Grande, Mato Grosso do Sul, Brasil; 2 Centre for Tropical Environmental and Sustainability Science and College of Science and Engineering, James Cook University, Cairns, Australia; 3 Falk School of Sustainability, Chatham University, Gibsonia, Pennsylvania, United States of America; 4 Universidade Estadual de Mato Grosso do Sul, Aquidauana, Mato Grosso do Sul, Brasil; 5 Odum School of Ecology, University of Georgia, Athens, United States of America; Universitat de Barcelona, SPAIN

## Abstract

Species loss from upper trophic levels can result in some major changes in community structure and ecosystem functions. Here, we experimentally excluded macroconsumers (e.g., fish and shrimp) in a Brazilian karst tropical stream during the dry season to investigate if their loss affected the accrual of calcium, dry mass (DM) and ash-free dry mass (AFDM) of sediment, benthic invertebrates, and chlorophyll-a. We found that the exclusion of macroconsumers decreased accrual of calcium. The absence of fish and shrimp may have promoted increased grazing by mayflies and snails in the electrified treatment as expressed by the depressed calcium accrual and shift in periphyton community composition. However, the exclusion of macroconsumers had no effect on DM and AFDM, chlorophyll-a, or total abundance of invertebrates. Our findings shed new light on the impact of macroconsumer loss and consequences for calcium accrual in karstic streams.

## Introduction

Human activities can disproportionally affect macroconsumers in the upper trophic levels of food webs (i.e., top predators). Loss of macroconsumers can result in rapid changes in community structure and ecosystem function [1–3]. In streams, macroconsumers (such as many species of fish and decapod crustaceans) can directly influence the accumulation, and transport of organic and inorganic sediments by disturbing substrates (bioturbation) and feeding at multiple spatiotemporal scales [4–10]. As a result, the presence of macroconsumers can reduce the rate of sediment accumulation on bottom substrates and can simultaneously limit the abundance and diversity of benthic algae and/or invertebrates that depend on specific substrates [5,11,12,13]. Although other experimental studies have reported the effects of the loss of macroconsumers and the importance of bioturbation on the accumulation of sediments in streams [4,14,15], the effects caused by macroconsumers on accrual of calcium carbonate-rich sediments are unknown.

In karstic streams, benthic algae and insect larvae such as Hydropsychidae and Chironomidae are known to affect the deposition of calcium carbonate and the formation of travertine or tufa sedimentary structures [16–19]. However, the potential for bioturbation is also particularly strong [16–17]. Invertebrate communities that include bioturbators can be altered both directly and indirectly by macroconsumers [20,21,22,23]. These top-down effects on benthic insects and algae can influence benthic primary production and food webs [21,24,25]. However, only a few studies have focused on tropical streams and the biotic relationships leading to sediment deposition [26,27].

Streams in karstic watersheds of the Neotropics are particularly threatened by human activities [28] where thin limestone soils provide minimal filtration to remove pollutants [28,29]. Typical of many tropical regions, the freshwater ecosystems in the Bodoquena Plateau of Central-West Brazil have high biodiversity but are at risk of ecological degradation [30]. Streams of the Bodoquena Plateau are threatened by the rapid conversion of natural vegetation to agriculture and pastures [30] and pressure from tourism [31,32]. Streams in the Bodoquena Plateau have numerous species of algivorous, omnivorous, and predatory fish and omnivorous shrimp and crabs [33,34] as well as aquatic insects and gastropods [35], including endemic and endangered species (e.g., *Ancistrus formoso* and *Trichomycterus dali*) [36].

These streams have complex hydrological connectivity with groundwater inflows due to the surrounding limestone-dominated watershed. Consequentially, the streams have high levels dissolved calcium and bicarbonate [37]. These inflows affect the growth and species composition of the periphyton and the abundance of diverse taxa of benthic invertebrates as observed in other karstic streams [38,39]. Previous studies on stream ecosystems in this region reported that the experimental exclusion of macroconsumers did not directly affect the benthic community and organic and inorganic sediment [34]. However, the rapid loss of natural cover in some parts of the Bodoquena Plateau watersheds and the potential loss of macroconsumers intensifies the need to determine the vulnerability of these streams to ecological change. In addition, the complex network of karst riverine systems is characterized by waterfalls formed by calcium carbonate deposition (travertine) that limit access by some fishes. In this context, the Bodoquena Plateau streams provide an ideal opportunity to examine if the loss of macroconsumers influences how species interactions affect sedimentary processes and benthic invertebrate communities that help to maintain clean waters.

The purpose of this study was to evaluate whether the presence of macroconsumers (e.g., fish and shrimp) affect the accumulation of calcium and organic sediments, as well as benthic invertebrate abundance, and biofilm production in the benthos of a representative karstic stream in the Bodoquena Plateau. We hypothesized that experimental exclusion of fish and shrimp would decrease bioturbation of sediments and increase accumulation of calcium-rich sediments. Additionally, we expected that the absence of predation would increase the abundance of grazing aquatic insects and gastropods. This increased grazing would decrease biofilm biomass (combinations of benthic micro-algae and microbes within a polysaccharide matrix). Our hypotheses were based on related research that concluded the absence of macroconsumers in stream ecosystems can increase the accumulation of inorganic sediment and organic material [5,15]. Biofilms provide energy to invertebrate consumers while also influencing sediment deposition and stability during base flows [10,40]. Studies of tropical streams in non-karstic ecosystems have demonstrated that the absence of macroconsumers can reduce the bioturbation activities by affecting the activity of other stream biota [5,15]. To test these hypotheses, we experimentally excluded macroconsumers and examined the effects of exclusion on sediment accrual, benthic invertebrates, and periphyton as dependent variables.

## Materials and methods

### Ethics statement

Permits of animal handling for this study were approved by the Ministério do Meio Ambiente (MMA), Instituto Chico Mendes de Conservação da Biodiversidade (ICMBio), Sistema de Autorização e Informação em Biodiversidade (SISBIO) under protocol number (7028–1) and authentication code (91743927). Permission to work at the National Park (Parque Nacional da Serra da Bodoquena) was granted by the ICMBio (Bonito, Mato Grosso do Sul, Brazil) that approved the protocols for this project. This study did not involve endangered or protected species.

### Study site

The study was conducted in a headwaters stream, Córrego Taquaral, located in the Bodoquena Plateau, Mato Grosso do Sul, Brazil (21°15'21.8" N, 056°21'20.4" W) during 12–26 June 2016. The Bodoquena Plateau is characterized by karstic watersheds and is one of the most extensive continuous karst aquatic systems in Brazil [41]. The Córrego Taquaral is a second- to third-order stream tributary of the Formoso River, which ultimately drains into the Upper Paraguay River basin and the Pantanal–the world’s largest and most biodiverse tropical wetland. Vegetation surrounding the study site consists of deciduous and semi-deciduous forests. The site is located at the transition between Cerrado (savanna) and Atlantic rainforest forest ecoregions, both considered hotspots of biodiversity [42]. Most of the Córrego Taquaral watershed is protected by the Parque Nacional da Serra da Bodoquena (PNSB) and the stream currently represents one of the reference areas of PNSB’s Long Term Ecological Research program (LTER) [43].

The Córrego Taquaral flows from an elevation of 400 to 358 m above sea level, ranges from 8 to 30 m wide, and was 50–70 cm deep during the study. Flow is groundwater fed and relatively uniform during the dry season when this experiment was conducted. Substrate consists primarily of sand as well as large boulders and cobble-sized inorganic sediments with travertine deposition. High calcium carbonate concentrations and elevated pH during photosynthesis result in the formation of numerous calcareous tufa formations, travertine waterfalls, and calcium carbonate deposits along pool and riffles downstream. The steep waterfalls are natural barriers to movements of some species of predatory fishes [44].

Due to the broad channel width (>20 m), the Córrego Taquaral possesses an open canopy and benthic algae (periphyton) provide the primary resource base for the food web. The periphyton assemblage is dominated by Chlorophyceae (*Stigeoclonium* sp.), diatoms, and cyanobacteria [34]. The benthic macroinvertebrate community is dominated by aquatic insects, primarily species of Ephemeroptera, Trichoptera, Diptera, Megaloptera, and Odonata [35]. Macroconsumers include insectivorous fish in four families [45–47]: Characidae (*Xenurobrycon macropus*), Crenuchidae (*Characidium zebra*), and Heptapteridae (*Rhamdia quelen*). In addition, there are algivous fish: Loricariidae (*Ancistrus* sp.) and (*Hypostomus* sp.) that feed on periphyton [47] and two decapod species, an omnivorous shrimp (*Macrobrachium brasiliense*) and a crab (*Sylviocarcinus australis*) that consume algae and insects [48].

### Stream attributes

Multiple physical and chemical parameters were recorded during the experiment to characterize the study site. Water quality parameters, including temperature 20.4°C (± 0.59; mean ± one standard deviation); conductivity (407 ± 6.90 μ.S^-1^); salinity (0.2 ± 0.005 ppt); pH (7.7± 0.27); and dissolved oxygen (4.0± 0.96 mg l^-1^) were measured once daily with a multi-parameter meter (HI 9828 Hanna Instruments, USA). Total nitrogen (TN; 1.21 mg l^-1^) and total phosphorus (TP; 0.10 mg l^-1^) were also recorded during the experiment. TN was measured using persulfate methods and cadmium reduction, while TP was measured using persulfate and with stannous chloride. Mean values were determined using spectrophotometry [49].

### Study design

**Fig 1:**
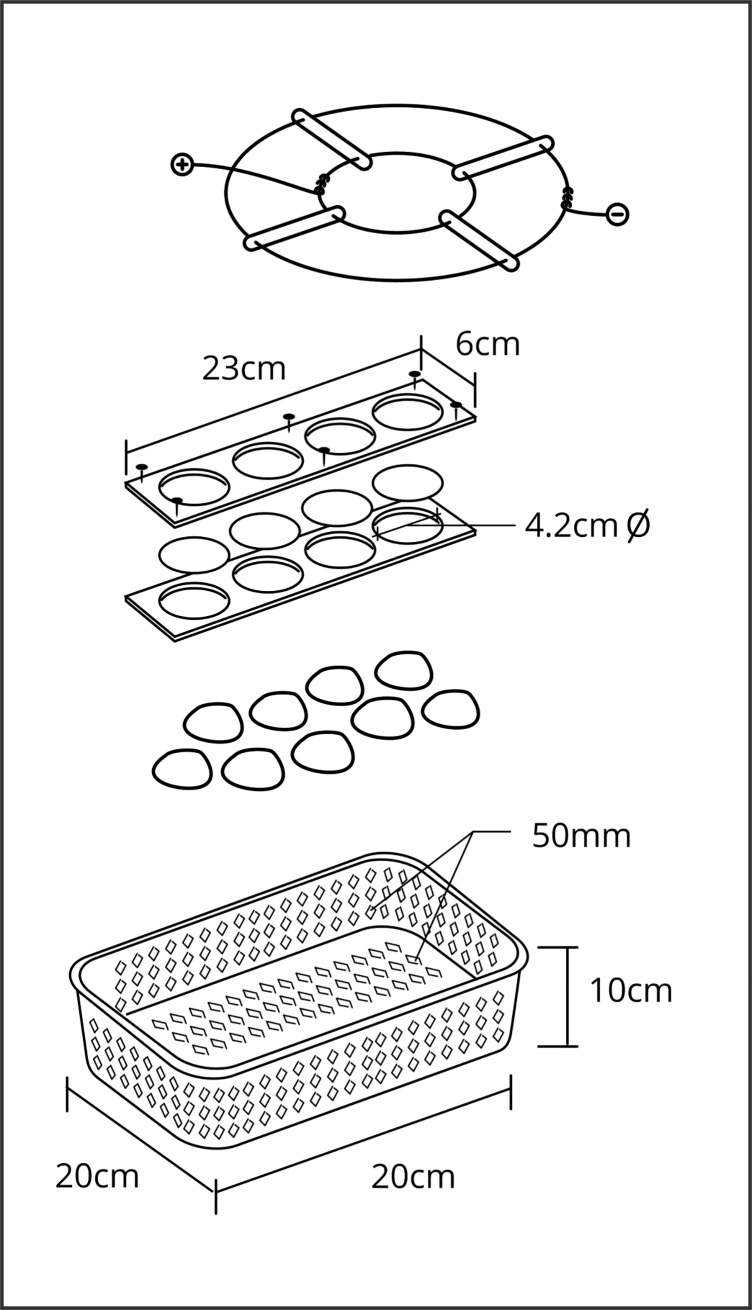
Diagram of the exclusion apparatus. Diagram of the materials used in exclusion experimental; one of the 15 baskets that contained stones for colonization by benthic invertebrates and periphyton colonization and glass fiber filters for sediment accumulation. Dimensions are indicated.

The experiment was conducted in the headwaters of the Córrego Taquaral stream during the early dry season. Our experimental design was a randomized complete block design with one treatment, an electrical fence exclusion of macroconsumers, and control samples. Replicate pairs of baskets were deployed in a large pool with depths of 60 cm. Ten paired substrate-filled baskets (five electrified and five control) were deployed for each section of the pool (lower, middle, and upper). Macroconsumer exclosures consisted of 20 × 20 × 10 cm plastic baskets with 50 mm mesh opening to maintain some limited flow through the rock substrates and with a 50 mm mesh in the bottom of the baskets (as previously recommended for use in the U.S. National Science Foundation’s STREON program in the National Ecological Observatory Network) [50,51]. The mesh size allowed smaller benthic invertebrates and microbes to colonize the chambers but prevented the larger macroconsumers from entering the enclosures. Iron rings that served as electrodes were attached to the top of the baskets using plastic zip ties (Fig 1). The pulsed electrical charge was produced by a 1.2 J livestock fence charger (LHR Manutenção e Montagem Ltd. Ribeirão Preto, São Paulo, Brazil), 110 μs of duration and 1.3 amperes, with power supplied by a 12 V battery [34,52]. All baskets were filled with 20–30 similarly sized (approximately 40 cm^2^), round limestone rocks to promote colonization of invertebrates (Fig 1). These rocks were acquired from a commercial (Lilu’s Black Floricultura, Campo Grande, Brazil) mining source of limestone to standardize the replicates and to ensure no prior biological colonization. Basket positions within each replicate were randomized with a minimum distance between baskets of 1 m. For additional details on the electric exclosure design, see [34]. The response of the animals to electric shock was immediately observable. No fish and shrimp entered the electrified treatments during approximately one hour of daily observation of the replicates to establish functional efficacy. Control baskets were constructed in the same manner as electrified baskets. However, these baskets were not connected to a fence charger and visual observations documented that macroconsumers entered the control baskets.

We standardized measurements of periphyton colonization and inorganic matter accumulation inside each basket by installing a 23 × 6 cm thick acrylic panel with four circular holes of 4.2 mm diameter (Fig 1). Each hole was fitted with glass fiber filters (0.45 μm porosity, 47 mm diameter) that acted as substratum for sediment precipitation as well as bioturbation (Fig 1); these filters have been used in other exclusion experiments in Brazil as substrate for growth of periphyton [53]. Filters were individually weighed prior to deployment. The acrylic panels with filters in each basket were located between the stones while the filters were positioned above the baskets (Fig 1).

### Processing of benthic ecological parameters

The experiment was conducted during 15 days. The duration of the experiment was based on our review of the literature and on our previous studies as well as being constrained by the variable duration of the dry season in this region. We expected that: (i) colonization time of benthic macroinvertebrates would be rapid in tropical streams [54,5,55], (ii) the baskets would be colonized by most groups of benthic invertebrates that occur in the region (e.g., Gastropoda, as well as larvae of Diptera, Ephemeroptera and Trichoptera) including different functional feeding groups such as diverse grazers and predators (Odonata larvae) [35]; and (iii) calcium accrual would be detectable on bare-rock substrates after 10 to 15 days.

Ten baskets randomly were collected on days 5, 10, and 15 of the experiment. Four filters from each board/basket were collected using a spatula to retain as much sediment as possible. Filters with sediments were placed in aluminum foil, individually marked, and dried for 1 h at 105°C in the laboratory to obtain dry mass (DM) then ashed for 2 h at 550°C and reweighed to estimate the ash-free dry mass (AFDM; i.e., organic matter). Total sediments deposited on filters were then calculated as DM and AFDM. We sampled the baskets for benthic invertebrates by disrupting the sediments by hand in front of a 0.5 mm mesh kick net positioned immediately downstream to catch any organisms that had colonized the stones. Benthic invertebrates were also sorted from the substrate in each basket and were preserved in 80% ethanol for subsequent identification in the laboratory. All insects were identified, usually to family, using taxonomic keys and original descriptions [56,57].

All stones in each basket were scraped and washed into a bucket to create a 200 ml slurry for analysis of primary producer mass in the form of chlorophyll-a. We used a syringe to extract a 50 ml subsample from the slurry for periphyton analysis. Each periphyton sample was obtained using pre-ashed glass fiber filters (0.45 μm porosity, 47 mm diameter). Chlorophyll-a was extracted from each filter in 10 ml 80% ethanol for 24 h in a freezer in the absence of light [58] and concentrations were measured using a spectrophotometer (model: Hach Dr 6000, Loveland, Colorado USA).

We selected 18 replicates, nine from each treatment, with four filters each to analyze calcium from the concentration of dry mass. Filters were macerated using acid digestion and peroxide of hydrogen (method 3050B) [59] and later analyzed using spectrophotometry of emission (method 3030.F in a SPECTRAA 220 FS) [60] to determine calcium accrual on filters.

### Statistical analyses

We applied repeated measures linear mixed or generalized linear mixed models to test for the effects of the exclosure treatment on all dependent variables of interest. For each statistical model, the stream section (lower, middle, or upper) was treated as a random block factor. Continuous dependent variables, including DM, AFDM, calcium, and chlorophyll-a were modeled using linear mixed models. Data were log-transformed (x+1, to preserve samples with zero values) to fit the assumption of normality when appropriate. For discrete dependent variables, such as the abundance and richness of benthic invertebrates, generalized linear models assuming Poisson-distributed data were derived also using the stream section as a random block factor. All models included day of trial and experimental status as independent variables. Models for dependent variables representing accrual on tiles included the abundance of scrapers and collector-gatherers as independent variables as well. Degrees of freedom for mixed models were estimated using the Satterthwaite approximation [61].

We also tested for differences in benthic invertebrate communities among treatments using permutational multivariate analysis of variance (PERMANOVA) on Bray-Curtis dissimilarities derived from log-transformed abundance data. To determine if these parameters changed over the course of the study, we assessed linear regression models of readings with time as an independent variable.

All analyses were performed in open-source software R [62]. Mixed models were developed using the lmerTest package [63] and PERMANOVA models were estimated using the package vegan [64].

## Results

Most physical and chemical measures, including pH and dissolved oxygen remained uniform throughout the study period (p>0.05), while temperature, conductivity, and salinity did increase linearly (p<0.05). These increases were likely due to the advancement of the dry season during the study period. The total phosphorus was within the range designated for unpolluted streams in the region [65].

**Fig 2:**
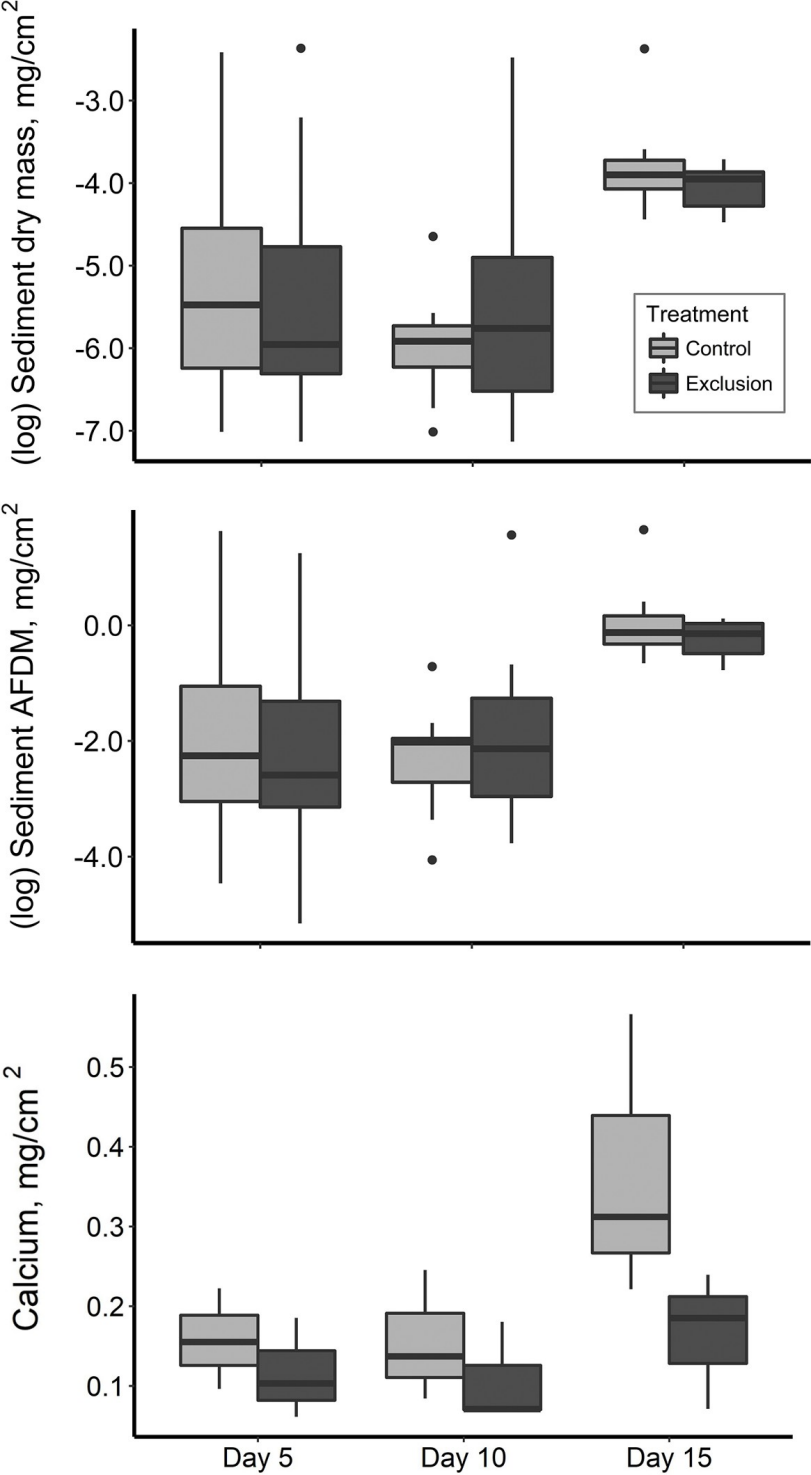
Differences in sediment accrual. Sediment DM (mg/cm^2^) (top panel), sediment AFDM (mg/cm^2^) (medium) and calcium (mg/cm^2^) (bottom panel) between control and exclusion from the three period of time. A linear mixed model suggested that the differences between control and treatment units were statistically significant by day 15.

We found a significant difference in patterns of calcium accrual between treatments, with an increase in the controls compared with the electrified treatments (Fig 2 and Table 1). The calcium accrual (based on dry mass) varied between treatments: control (0.00057–0.00008 mg/cm^2^) and electrified (0.00024–0.00006 mg/m^2^). However, the exclusion of macroconsumers had no effect on DM (sediment) or AFDM (organic material) (Fig 2 and Table 1). The mean sediment dry mass was similar between control (0.091 mg/cm^2^) and electrified treatments (0.104 mg/cm^2^).

**Table 1:** Statistical parameters associated with linear mixed and generalized linear mixed models. Coefficients are provided only for statistically significant model terms and represent the factorial of introducing an electrified exclosure in the treatment parameter.

		Day		Scraper/collector-gatherer abundance		Exclosure treatment		
Parameter	Units	z-value	p-value	z-value	p-value	z- value	p-value	Coefficient
**Calcium**	mg/cm^2^	1.96	0.005	0.40	0.692	2.20	0.028	-1.0×10^−4^
**(log) Dry mass**	mg/cm^2^	5.08	<0.001	2.08	0.040	0.13	0.893	
**(log) AFDM**	mg/cm^2^	1.90	0.066	1.76	0.082	0.14	0.893	
**Chlorophyll-a**	μg /cm^2^	2.04	0.051	1.70	0.101	0.24	0.811	
**Benthic invertebrate abundance**	N	7.97	<0.001			0.60	0.547	
**Benthic invertebrate richness**	N	3.47	<0.001			0.00	0.999	

**Fig 3:**
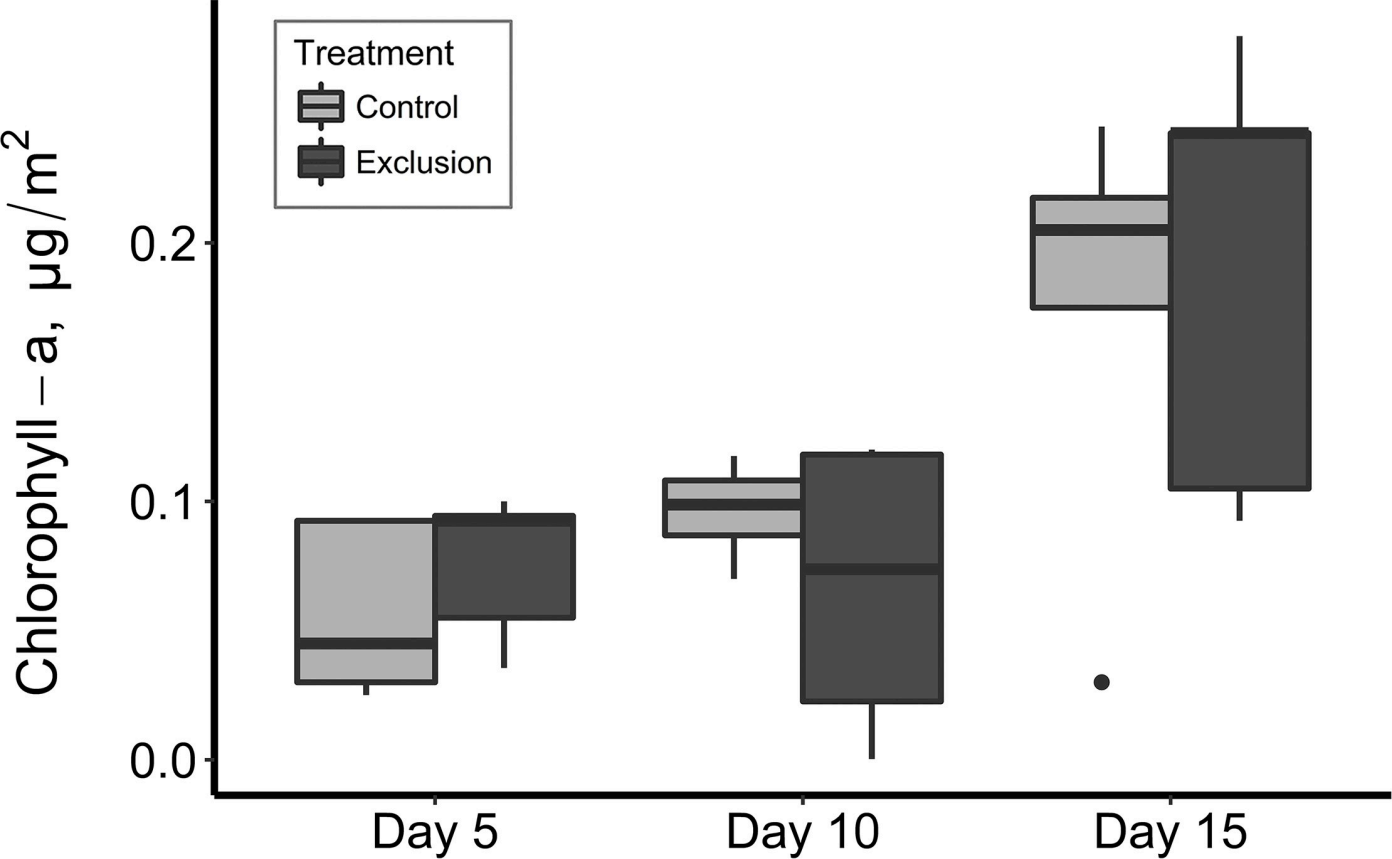
Differences in chlorophyll-a concentrations (μg/cm^2^) between control and treatment baskets from the three period of time. The data are calculated from linear mixed models and were treated from random block factor. A linear mixed model suggested that chlorophyll-a values did not indicate significantly differ between treatments, although increases over time were statistically significant.

There were no differences in chlorophyll-a concentration between treatments (Fig 3), suggesting that exclusion of macroconsumers did not influence the abundance of grazing invertebrates that would decrease periphyton growth on substrates inside of baskets.

**Fig 4:**
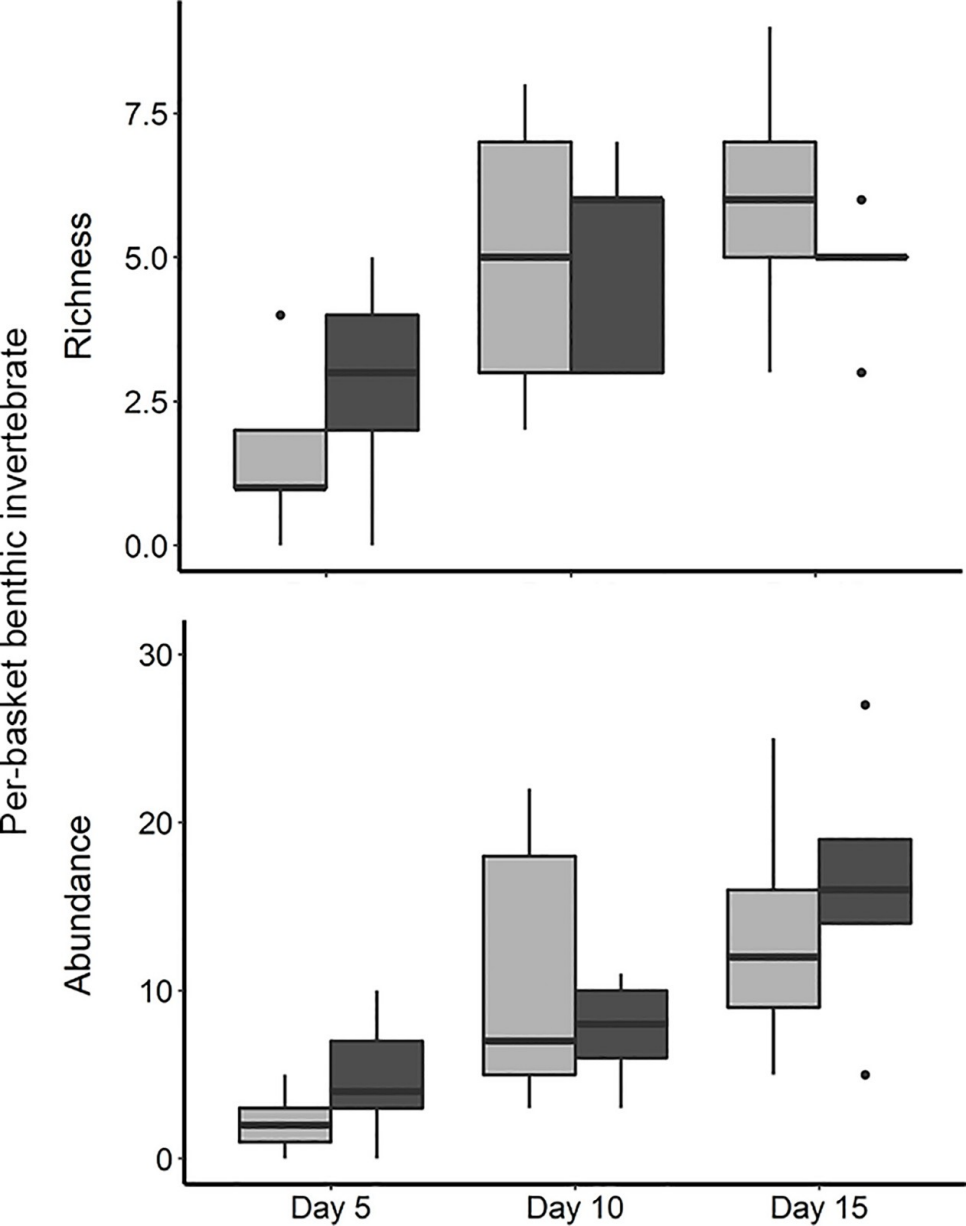
**Total richness (top panel) and abundance of benthic invertebrates (bottom panel) between control and treatment baskets from the three period of time.** Data were compared using generalized linear models assuming Poisson distributions. Invertebrate abundance and richness did not significantly differ between treatments.

We observed no significant differences between treatments in the total richness and abundance of invertebrates colonizing the stony substrata (Fig 4 and Table 1). We collected 276 benthic invertebrates from the baskets representing 19 families of mostly aquatic insect larvae (S1 Table). The total abundance of taxa in substrata among treatments was 133 (control) and 143 (electrified treatments). The three most dominant benthic invertebrates were insects: Chironomidae (Chironominae), Trichoptera (Leptoceridae), and Ephemeroptera (Leptophlebiidae). There were no differences in abundance of insects between treatments. The scrapers and collector-gatherer (i.e., taxa feeding on periphyton) decreased dry mass and appeared to affect AFDM and chlorophyll-a, although these effects were not significant at = 0.05 (Table 1). Scraper and collector-gatherer abundance did not affect calcium accrual.

Community composition, as assessed by a PERMANOVA model, was unrelated to treatment (F = 1.3, p = 0.219) or an interaction term between treatment and time (F = 0.8, p = 0.665) (Table 1).

## Discussion

Our findings suggest that excluding macroconsumers did not directly affect the abundance of benthic invertebrates, chlorophyll-a concentration or dry mass, and AFDM. Previous studies in the Bodoquena Plateau during the dry season and in tropical streams of Hong Kong have also reported that the absence of macroconsumers did not increase accumulation of sediments, periphyton accrual, or benthic invertebrate abundance [14,34,66].

The most interesting finding of our study was the increased accumulation of calcium in the presence of macroconsumers. The changes in calcium accumulation could be related to both direct and indirect effects of macroconsumers on trophic interactions. The indirect trophic interactions were likely associated with changing the behavior of some benthic invertebrates, particularly the insect larvae associated with travertine formation [16–19]. The presence of macroconsumers can influence the movement behavior of the benthic invertebrate prey that decreases their foraging and bioturbation, which can increase calcium accrual in the control treatment. However, this possible change in behavior of grazers did not alter algal and sediment biomass in our study. One potential explanation for a lack of this effect is the potential interaction among different growth rates among the algae species. A previous study [34] demonstrated that the composition of the algae changed when the macroconsumers were experimentally excluded. The growth rates and growth forms of the algae were likely influenced by benthic invertebrates as well as by calcium accrual [39]. These compositional changes in the algal assemblage could include compensatory mechanisms that resulted in no effect on the total amount of chlorophyll-a.

Benthic stream invertebrates have behavioral and morphological adaptations to avoid predators [67], especially in highly transparent karst water where benthic invertebrates are vulnerable to visually oriented predators [68]. Scraping mayflies, for example, commonly reduce foraging activities in the presence of predatory fish [69,70]. Although mayfly (e.g., Leptophlebiidae) abundance did not vary among treatments, a dominant grazer *Simothraulopsis* [71] occurred in all baskets and may have responded to the presence of macroconsumers by decreasing their grazing. As our experiment did not control for predator chemical clues, which were present in both control and exclusion treatments, it is unlikely that this indirect chemical-communication effects reported in other studies [72,73] can account for the differences we found. We speculate that the response of grazers in clear-water streams, like those grazers in streams in Bodoquena Plateau, involves visual cues and indirect effects on behavior such as crevice seeking.

Karstic streams are rich in dissolved calcium carbonate, carbon dioxide, and bicarbonate. When the dissolved carbon dioxide is used in photosynthesis, pH increases and deposition of calcium carbonate increases among algae and macrophytes in these streams [16,17]. This formation of calcium carbonate can increase transparency that influences foraging behavior of predators and their prey [33]. Insect larvae and microorganisms are strongly associated with precipitation and formation of travertine in these travertine-producing ecosystems [17,74]. Consequently, the presence of predators and omnivores can directly or indirectly affect their prey’s foraging and bioturbation [75,73]. Such changes of behavior likely increased the amount of calcium in the control treatment. Alternatively, the presence of omnivores and algivores could have changed the composition of algal community [34] and increased the pH that led to more deposition of calcium on the filters in the baskets [39].

Aquatic insect larvae such as the Hydropsychidae and Chironomidae build retreats (cases) using sediment particles or construct silken nets for filtering suspended organic particles [18,74]. During calcium carbonate precipitation their cases can be colonized by cyanobacteria that remove CO_2_ and increase pH that further enhances the accrual of calcium carbonate [17]. Although insect abundance in our study did not vary with respect to treatments, Trichoptera in the family Leptoceridae build tubular retreats and Chironomid build nets that are known to increase calcium carbonate deposition [18]. These were one of the most abundant insect taxa that occurred in the baskets. Some of these taxa would likely respond to the presence of macroconsumers by reducing their feeding and bioturbation activity that led to increasing the processes of encrustation and elevated the calcium accrual.

In our study, we found a low abundance of benthic invertebrates in both treatment and control baskets when compared to other experimental studies in tropical streams [52]. Calcium carbonate deposition has also been suggested to reduce invertebrate biomass and diversity [38,76]. Specimens of Chironominae, Leptophlebiidae, Mollusca, Trichoptera, and Odonata remained the most abundant insect groups in both treatment and control baskets. The larger (≥ 3.0 cm long) ampullarid and planorbid snails may have increased grazing while also reducing the calcium accumulation in the electrified treatment. The abundance of snails was also observed in travertine formation in other streams [76].

Calcium carbonate deposition is potentially related to many ecological attributes in karst stream ecosystems. For instance_,_ calcium carbonate-depositing portions of some karst streams are known to provide microhabitats and high biodiversity for algae and insect larvae [77], and other organisms [78]. This study is the first to experimentally document the importance of the presence of macroconsumers (e.g., fish and shrimp) on calcium accrual. Our findings shed new light on the impact of species loss on karstic streams and consequences for calcium dynamics in streams.

## Supporting information

S1 Table Supporting data. (PDF)
