# Supplementary material for: Effects of macroconsumers on benthic communities: Rapid increases in dry-season accrual of calcium in a tropical karst stream
Source: PLoS One. 2018 Dec 21;13(12):e0209102. doi: 10.1371/journal.pone.0209102 (PMC6303065; doi:10.1371/journal.pone.0209102)
Supplement: S1 Table — (PDF) [file pone.0209102.s001.pdf]

S1 Table 1. Results of invertebrate community in the control and exclusion treatments in June 2016 in the Taquaral stream, Brazil.

		Treatment	
**Order**	**Family**	**Control**	**Exclusion**
Insecta			
Coleoptera	Elmidae	5	9
	Hydrophilidae	1	1
Diptera	Ceratopogonidae	11	5
	Chironominae	27	31
	Orthocladiinae	1	
	Tabanidae	2	2
	Tanypodinae	7	8
Ephemeroptera	Baetidae	1	5
	Caenidae	6	3
	Leptophlebiidae	17	27
Lepidoptera	Pyralidae		1
Odonata	Aeshnidae		1
	Coenagrionidae		1
	Gomphidae	3	6
	Libellulidae	1	
Plecoptera	Gripopterygidae	1	
Trichoptera	Calamoceratidae		2
	Helicopsychidae	8	1
	Hydropsychidae	5	4
	Leptoceridae	27	25
Gastropoda			
Caenogastropoda	Ampullariidae		1
	Hydrobiidae	5	
Pulmonata	Planorbidae	5	10
